# Pink1/PARK2/mROS-Dependent Mitophagy Initiates the Sensitization of Cancer Cells to Radiation

**DOI:** 10.1155/2021/5595652

**Published:** 2021-07-06

**Authors:** Lei Yu, Xiangshan Yang, Xin Li, Lijing Qin, Weiqiang Xu, Hongli Cui, Zhen Jia, Qiang He, Zhicheng Wang

**Affiliations:** ^1^NHC Key Laboratory of Radiobiology (Jilin University), School of Public Health, Jilin University, Changchun 130021, China; ^2^Department of Radiotherapy, Second Hospital of Jilin University, Changchun 130041, China; ^3^Department of Radiotherapy and Medical Technology, Jilin Cancer Hospital, Changchun 130000, China

## Abstract

Autophagy plays a double-edged sword for cancer; particularly, mitophagy plays important roles in the selective degradation of damaged mitochondria. However, whether mitophagy is involved in killing effects of tumor cells by ionizing radiation (IR) and its underlying mechanism remain elusive. The purpose is to evaluate the effects of mitochondrial ROS (mROS) on autophagy after IR; furthermore, we hypothesized that KillerRed (KR) targeting mitochondria could induce mROS generation, subsequent mitochondrial depolarization, accumulation of Pink1, and recruitment of PARK2 to promote the mitophagy. Thereby, we would achieve a new strategy to enhance mROS accumulation and clarify the roles and mechanisms of radiosensitization by KR and IR. Our data demonstrated that IR might cause autophagy of both MCF-7 and HeLa cells, which is related to mitochondria and mROS, and the ROS scavenger N-acetylcysteine (NAC) could reduce the effects. Based on the theory, mitochondrial targeting vector sterile *α*- and HEAT/armadillo motif-containing protein 1- (Sarm1-) mtKR has been successfully constructed, and we found that ROS levels have significantly increased after light exposure. Furthermore, mitochondrial depolarization of HeLa cells was triggered, such as the decrease of Na^+^K^+^ ATPase, Ca^2+^Mg^2+^ ATPase, and mitochondrial respiratory complex I and III activities, and mitochondrial membrane potential (MMP) has significantly decreased, and voltage-dependent anion channel 1 (VDAC1) protein has significantly increased in the mitochondria. Additionally, HeLa cell proliferation was obviously inhibited, and the cell autophagic rates dramatically increased, which referred to the regulation of the Pink1/PARK2 pathway. These results indicated that mitophagy induced by mROS can initiate the sensitization of cancer cells to IR and might be regulated by the Pink1/PARK2 pathway.

## 1. Introduction

Currently, radiotherapy remains the chief strategy for cancer therapy and draws great attention in the medical field. However, the radioresistance of a large portion of tumors commonly leads to the failure of tumor treatment or tumor recurrence [1, 2]. Therefore, to overcome the radioresistance or to enhance the radiosensitivity is important [3]. Some studies have showed that autophagy strengthens the anticancer effects of radiotherapy on patients with oral squamous cell carcinoma [4] and sensitizes cancer cells to radiotherapy [5].

Autophagy is a basic process of catabolism of cellular components, such as the cytosol, organelles, and protein aggregates. In the last few years, the biological importance and molecular mechanisms have been extensively studied [5]. Autophagy involves the formation of double-layered membrane structures called autophagosomes which are trafficked to lysosomes in a dynein-dependent manner along microtubules. Autophagosomes can contain virtually any cytoplasmic element, including cytosolic proteins and various membranous organelles, mitochondria, endoplasmic reticulum, and peroxisomes [6]. Autophagy has two primary opposing functions in response to stress induced by radiation in tumor cells. Autophagy contributes to maintain cellular homeostasis, which is made to be cytoprotective function; however, as a type II programmed cell death, autophagy also plays important roles in improving radiosensitivity of cancer [7]. Autophagy has played a double-edged sword in the initiation, development, and metastasis of cancer.

Many recent findings have revealed that specific types of autophagy referred with selective degradation of peroxisomes (pexophagy), endoplasmic reticulum (ERphagy), mitochondria (mitophagy), ribosomes (ribophagy), or even nucleophagy [8, 9]. Mitochondria are essential organelles for energy production, cell survival, cell death, and cell signaling [10]. Mitochondria are also one of the main sites for production of mitochondrial reactive oxygen species (mROS) in the cell. Mitochondrial damage caused by cellular oxidative stress will eventually lead to cell death [11]. Mitophagy initiated by damaged mitochondria could serve as an alternative and complementary approach to initiating the death of cancer cell for potential cancer therapy [12]. The mitochondrion and its contents are prone to oxidative damage resulting from mROS produced in the matrix. Mitochondria selective autophagy-lysosomal degradation is mainly governed by posttranslational modifications of phosphatase and tensin homolog- (PTEN-) induced putative protein kinase 1 (Pink1) and Parkinson disease protein 2 (PARK2) [13–15]. Membrane depolarization induced by mROS stabilizes Pink1 on mitochondrial outer membrane and recruits PARK2, an E3-ubiquitin ligase, to mitochondria. PARK2 ubiquitinates mitochondrial substrates, and ubiquitin chains serve as a signal for both proteasome degradation and mitophagy [15, 16]. Thus, mitophagy mediated by mROS may be equally effective in anticancer therapy.

The mROS are produced by a free radical chain reaction, and they are directly involved in oxidative damage to lipids, proteins, and nucleic acids as well as depleting antioxidants in cancer cells [17, 18]. Bulina et al. developed a photosensitizer protein called KillerRed (KR), which is a dimeric red fluorescent protein with excitation and emission maximum at 585 and 610 nm, respectively [19, 20]. Photosensitizers are molecules producing ROS during photoreaction and are widely used in photodynamic therapy (PDT) for numerous cancer and certain noncancerous disease [21, 22]. KR can be expressed in a spatial and temporal manner regulated by choosing an appropriate promoter or fusing with a protein of interest and a subcellular localization signal. The subcellular localization of the KR protein is of special importance, because it determines the localization of the damage. KR protein can be used for the inactivation of light-induced protein, killing specific cell populations *in vivo*, and studying intracellular local oxidative stress [23–26].

In this study, we are aiming to figure out the roles of mitochondria and mROS in the regulation of autophagy induced by ionizing radiation (IR). Furthermore, the N-terminal mitochondrial-targeting sequence (MTS) of sterile *α*- and HEAT/armadillo motif-containing protein 1 (Sarm1) was used to mediate downstream KR (Sarm1-mtKR) to express in mitochondria [27]; then, mROS was induced by light inactivation of KR protein, to explore the mitochondrial dysfunction and mitophagy by light and IR, and mechanisms of the Pink1/PARK2 pathway, to provide a new insight for efficacy of combination of radiotherapy and PDT.

## 2. Materials and Methods

### 2.1. Cell Lines, Reagents, and Antibodies

Human breast cancer cell line MCF-7, human cervical cancer cell line HeLa, and African green monkey kidney cell line COS-7 were obtained from the American Type Culture Collection (ATCC) and cultured in Dulbecco's modified Eagle's medium (DMEM, Gibco, Grand Island, NY, USA), supplemented with 10% fetal bovine serum (MRC, Jiangsu, China) and 1% penicillin/streptomycin (Invitrogen, Carlsbad, CA, USA). Na^+^K^+^- and Ca^2+^Mg^2+^-ATPase kits were purchased from Nanjing Jiancheng Bioengineering Institute, China, MitoTracker Green dye kit and mitochondrial separation reagents were purchased from Beyotime® Biotechnology, Hangzhou, China, and mitochondrial respiratory complex I and III detection kits were purchased from Solarbio® Life Science, Beijing, China. Monodansylcadaverine (MDC), N-acetyl-L-cysteine (NAC), Rhodamine123 (Rh123), and 2′,7′-dichlorodihydrofluorescein diacetate (DCFH-DA) were purchased from Sigma-Aldrich (St. Louis, MO, USA). Anti-COX IV, anti-*β*-actin, and anti-GAPDH were purchased from Santa Cruz, CA, USA; anti-voltage-dependent anion channel 1 (VDAC1), anti-heat-shock protein 60 (HSP60), anti-Pink1, and anti-PARK2 were purchased from Bioworld Technology, Inc., USA; and anti-microtubule-associated protein 1 light chain 3 (LC3), anti-p62, and anti-Tom20 were purchased from Cell Signaling Technology, Danvers, MA, USA. IgG-HRP-conjugated secondary antibody was purchased from ImmunoWay, Plano, TX, USA.

### 2.2. Construction of Vectors Targeting Mitochondria

Because KR is inactivated by light and is replaced by another red fluorescence protein mCherry (no inactivation by light) to study intracellular localization, vectors of Sarm1-mtmCherry and Sarm1-mtKR were constructed by recombinant technique. The following primers were used: Sarm1-MTS: 5′-AAGGAAAAAAGCGGCCGCAATGGTCCTGACGCTG-3′ (F), 5′-CGGAATTCCCGATCGGCGCCCGGCCGTG-3′ (R); mCherry: 5′-GGAATTCGCCACCATGGTGAGCAAGGG-3′ (F), 5′-CGGGATCCTTACTTGTACAGCTCGTCCATG-3′ (R); KR: 5′-GGAATTCATGGGTTCAGAGGGC-3′ (F), 5′-CGGGATCCCTAGATCTCGTCG-3′ (R). Samr1, mCherry, and KR fragments were amplified with PCR using a Q5 High-Fidelity DNA polymerase kit purchased from EB, Beverly, MA, USA. Plasmids of plxsp-TetA-mCherry and plxsp-TetA-KillerRed kindly given by Dr. Shen from Cancer Institute of New Jersey, USA, and pGw1-myc-Sarm1 plasmid purchased from Addgene, Cambridge, MA, USA, were used as templates.

### 2.3. Cell Transfection, Fluorescence Microscope Observation

Plasmids of Sarm1-mtmCherry were transfected into HeLa and COS-7 cells with Hieff Trans™ Liposomal Transfection Reagent purchased from Shanghai YESEN Biotechnology Co., Ltd. for 30 h; then, coverslips were taken out, fixed with 4% paraformaldehyde in PBS for 10 min at room temperature (RT), and permeabilized and blocked with sealing fluid (0.3% Triton X-100 and 2% BSA in PBS) for 1 h at RT. Cells were incubated with COX IV antibody overnight at 4°C, followed by incubation with secondary antibodies (green fluorescence) for 1 h at 37°C. The coverslips were mounted onto microscope slides, and mCherry and COX IV expressions were observed under a fluorescence microscope. HeLa and MCF-7 cells were treated with NAC and 8 Gy IR (dose rate = 1.0 Gy/min, X-RAD 320iX machine purchased from Precision X-ray, Inc., USA), at 24 h post-IR, and coverslips were taken out and washed with PBS twice. Cells were labeled with a 200 nM MitoTracker Green in DMEM for 30 min at 37°C. Mitochondria were observed with a fluorescence microscope.

### 2.4. ROS Content Detection with DCFH-DA Staining

MCF-7 and HeLa cells were treated with 1 mM NAC and IR and stained with DCFH-DA (10 *μ*M), and ROS contents were analyzed by Flow Cytometry (FCM, Becton, Dickinson and Company, Franklin Lakes, NJ, USA). Additionally, HeLa cells were transfected with empty vector and Sarm1-mtKR plasmids for 30 h and exposed to visible light for 10, 30, and 60 min, respectively; then, at 10, 30, and 60 min after exposure, DCFH-DA was added into cells. Finally, the mean fluorescence intensity (MFI) was detected by the Cytation™ 3 Cell Imaging Multi-Mode Reader System (BioTek, Winooski, Vermont, USA). The experiment was performed in triplicate.

### 2.5. Flow Cytometry (FCM)

Autophagic rate and mitochondrial membrane potential (MMP) were measured with FCM. MCF-7 and HeLa cells were treated with 1 mM NAC and IR, stained with 50 mM MDC for 30 min at 37°C, then fixed with 4% paraformaldehyde for 10 min, and washed with PBS, and autophagic rate was analyzed by FCM. HeLa cells were transfected with empty vector and Sarm1-mtKR plasmids, respectively, after 30 h, cells were exposed to visible light and IR, and autophagic rate was measured using methods described as above. Additionally, the cells treated with light and IR were resuspended at 12 h post-IR, and Rh123 was added into the cells to yield final concentrations of 5 *μ*M for detecting MMP by FCM.

### 2.6. Mitochondrial Extraction

After transfection with empty vector and Sarm1-mtKR plasmids, HeLa cells were treated with light and IR. At 24 h post-IR, cells were collected at ×200 g for 5 min, added with 3 ml mitochondrial separation reagents with PMSF, and put on ice for 10 min. The cell homogenate was transferred into a glass homogenizer, performed for 30 min, and centrifuged at ×600 g at 4°C for 10 min. The suspension was transferred to another tube and centrifuged at ×11000 g at 4°C for 10 min. When the suspension was removed after centrifugation, the mitochondria were obtained. Then, the mitochondrial proteins were extracted and quantitatively determined.

### 2.7. Detection of Na^+^K^+^- and Ca^2+^Mg^2+^-ATPase and Mitochondrial (mito-) Respiratory Complex I and III Activities

After mitochondrial protein concentrations were determined, Na^+^K^+^- and Ca^2+^Mg^2+^-ATPase and mito-respiratory complex I and III activities were measured using biochemical assay kits according to manufacturer's protocol, and a spectrophotometer (Beckman, USA) with 636 nm, 340 nm, and 550 nm excitation wavelengths was used, respectively. There were 4 replicate wells per group, and the experiment was performed in triplicate.

### 2.8. Quantitative Real-Time PCR (qRT-PCR)

After transfection with empty vector and Sarm1-mtKR plasmids, HeLa cells were treated with light and IR. At 24 h post-IR, total RNA was extracted with TRIzol reagents (Invitrogen, Carlsbad, CA, USA), and the complementary DNA (cDNA) was synthesized using a high-capacity reverse transcription kit (Takara Bio Inc., Japan). GADPH: 5′-ACCACAGTCCATGCCATCAC-3′ (F), 5′-TCCACCACCCTGTTGCTGTA-3′ (R); Pink1: 5′-GGAGGAGTATCTGATAGGGCAG-3′ (F), 5′-AACCCGGTGCTCTTTGTCAC-3′ (R); Tom20: 5′-GGTACTGCATCTACTTCGACCG-3′ (F), 5′-TGGTCTACGCCCTTCTCATATTC-3′ (R) were used for mRNA detection. The qRT-PCR reaction was performed and analyzed (Bio-Rad, Hercules, CA, USA) according to the SYBR® Premix Ex Taq™ II kit (Takara Bio Inc., Japan) protocol.

### 2.9. Western Blot (WB)

After total and mitochondrial proteins were extracted and quantified, 40 *μ*g proteins were separated by SDS-PAGE (10% resolving gel, 5% stacking gel) and transferred to Nitrocellulose (NC) membrane (200 mA, 1.5 h; Merck Millipore, Billerica, MA, USA). The membrane was blocked with 5% nonfat dry milk and incubated with diluting solution of the primary antibodies overnight at 4°C. Anti-LC3, anti-VDAC1, anti-HSP60 and anti-p62, anti-Pink1, anti-PARK2 and anti-Tom20, anti-GAPDH, and anti-*β*-actin antibodies were used for WB. After washing with TBST, the membranes were incubated with IgG-HRP-conjugated secondary antibody for 1.5 h at RT. Finally, the membranes were identified using a chemiluminescence detection system (ECL detection kit, Santa Cruz, CA, USA). The films were scanned for gray scale ratio analysis.

### 2.10. Statistical Analysis

All statistical analyses were performed using SPSS version 24.0 (SPSS Inc., Chicago, IL, USA). The results were presented as mean ± SD and subjected to one-way ANOVA followed by Student's *t*-test, and *P* < 0.05 was considered to be statistically significant.

## 3. Results

### 3.1. Mitochondria and mROS Regulate Autophagy by IR in HeLa and MCF-7 Cells

**Figure 1:**
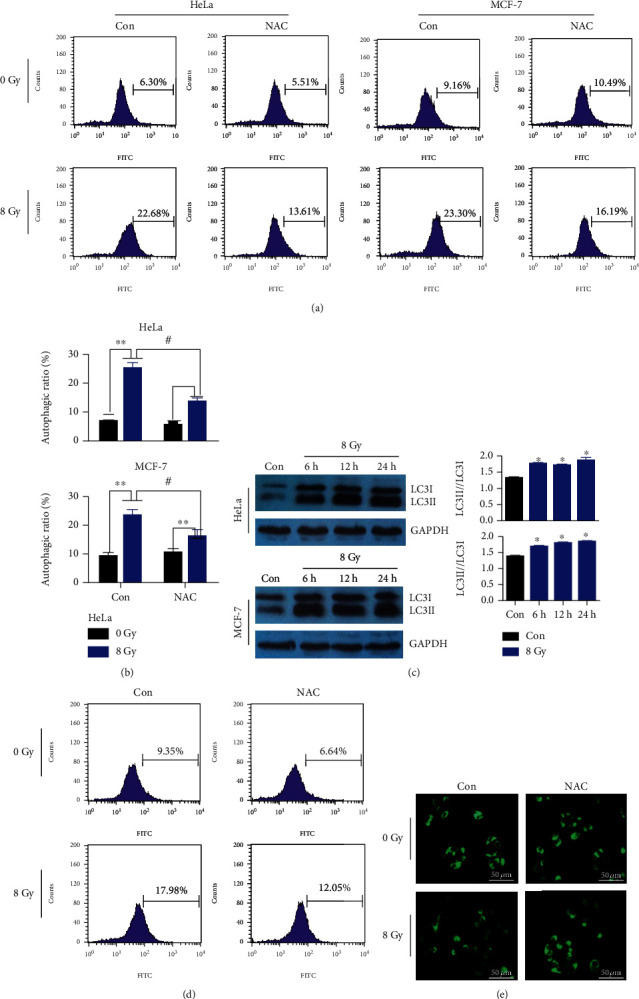
Changes of autophagy, mROS content, and mitochondrial impairment in HeLa and MCF-7 cells. (a) After treatment with 8 Gy IR and NAC (1 mM), representative pictures of autophagy were measured with MDC staining by FCM. (b) Autophagic rates were analyzed by FCM. (c) Western blot analysis of LC3, GAPDH protein was used for loading control, and LC3II/LC3I was quantified. (d) ROS contents were detected by DCFH-DA staining (10 *μ*M) and analyzed by FCM after 8 Gy IR and NAC (1 mM) treatment. (e) The mitochondrial mass was detected by MitoTracker Green (200 nM) staining. Scale bar, 50 *μ*m. Bars represent mean ± SD of triplicate measurements. ^∗^*P* < 0.05 and ^∗∗^*P* < 0.01, versus 0 Gy; ^#^*P* < 0.05, versus control.

HeLa and MCF-7 cells were treated with NAC and IR (8 Gy), and the autophagic rate was measured with MDC staining by FCM. The results showed that IR induced autophagy, while NAC decreased the IR-induced changes (Figures 1(a) and 1(b)). LC3 protein was used as an autophagic marker, and the LC3II/LC3I increased with time prolongation in both cell lines, suggesting that autophagic death has been induced by IR (Figure 1(c)). Furthermore, to evaluate the effects of mitochondria on regulating the autophagy, the mROS and mitochondrial damage were detected in MCF-7 cells. As shown in Figure 1(d), treatment with IR increased the ROS contents, while antioxidant NAC decreased them in cells. In addition, MitoTracker Green staining showed that IR induced mitochondrial impairment, NAC reduced the impairment in MCF-7 cells (Figure 1(e)), and the similar impairment in HeLa cells was demonstrated in the study by Chen et al. [28]. These data indicated that IR-induced mROS had effects on mitochondrial damage and then initiated the cell autophagy, while antioxidant NAC could protect the mitochondria from damage induced by IR.

### 3.2. Construction and Targeting Identification of Mitochondrial Targeting Vectors (Sarm1-mtKR)

**Figure 2:**
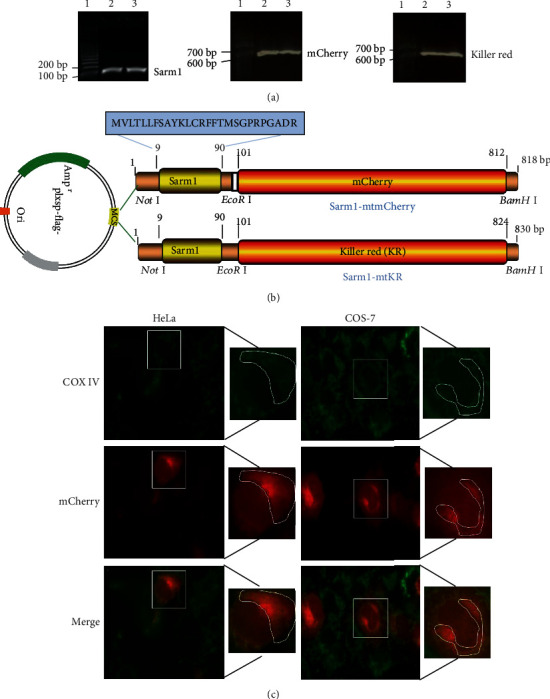
Construction of mitochondrial targeting vector and localization. (a) PCR products of Sarm1, mCherry, and KR. Lane 1 was 100 bp DNA marker, and lanes 2 and 3 were PCR amplification products. (b) Schematic diagram of Sarm1-mtmCherry and Sarm1-mtKR vectors: MTS (Sarm1) was cloned into empty vector (plxsp-flag) (Not I and EcoR I sites), and mCherry and KR were cloned into plxsp-flag-Sarm1-MTS (EcoR I and BamH I sites). (c) The expressions of COX IV (green) and mCherry (red) were observed after COS-7 and HeLa cells were transfected with Sarm1-mtmCherry plasmids, ×200.

PCR amplification products of Sarm1 (MTS), mCherry, and KillerRed (KR) were separated with DNA gel, and the fragment length was consistent with the prediction (Figure 2(a)). The Sarm1, mCherry, and KR fragments were inserted into plxsp-flag vector (empty vector) to construct plxsp-flag-Sarm1-mCherry (Sarm1-mtmCherry) and plxsp-flag-Sarm1-KR (Sarm1-mtKR), respectively (Figure 2(b)). All of the constructed plasmids have been sequenced to verify that the clones had the correct sequence. Sarm1-mtmCherry plasmids were transfected into COS-7 and HeLa cells; as shown in Figure 2(c), the mitochondrial tracker COX IV clearly expressed in the mitochondria; and at the same time, red fluorescence protein mCherry also specifically localized to the same site. Hence, these data indicated that the MTS of Sarm1 had mitochondrial targeting characteristics.

### 3.3. ROS Induced by Sarm1-mtKR Exposed to Visible Light

**Figure 3:**
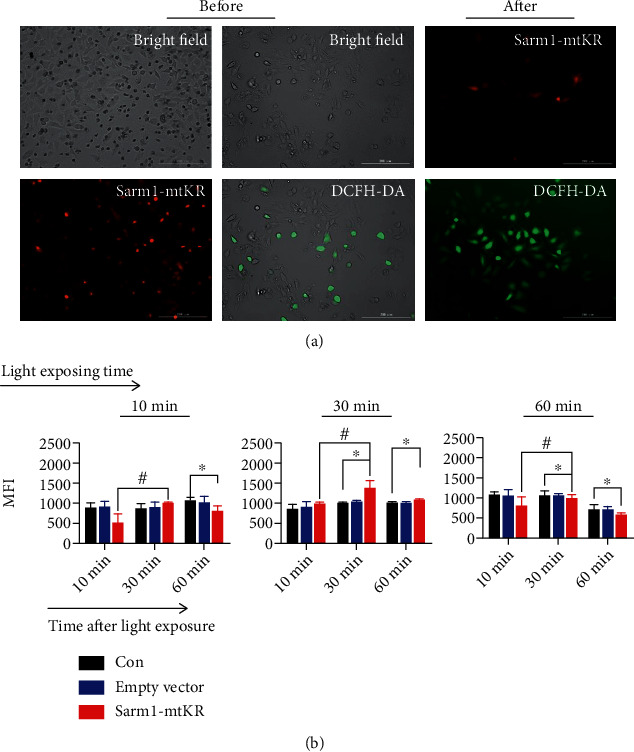
ROS content detection by DCFH-DA staining in HeLa cells. (a) Representative images of KR before and after light exposure for 30 min, and cells were stained by DCFH-DA (green), scale bars, 200 *μ*m. (b) The changes of MFIs at different time postlight exposure for 10, 30, and 60 min, respectively. Bars represent mean ± SD of triplicate measurements. ^∗^*P* < 0.05, versus control, ^#^*P* < 0.05, versus 10 min after light exposure.

KR protein was inactivated under visible light status, and then, ROS was produced. In order to explore the regularity of ROS production induced by Sarm1-mtKR, after plasmid transfection, cells were exposed to visible light for 10, 30, and 60 min, respectively. As shown in Figure 3(a), before light exposure, there were a large number of red cells and very few green cells; after light exposure, red cells decreased and green cells increased, indicating ROS production. MFIs reached for maximum value at 30 min post-10 or post-30 min light exposure (Figure 3(b)). These results indicated that light exposure caused the KR protein inactivation to increase the mROS production.

### 3.4. Mitochondrial Dysfunction Caused by mROS from Sarm1-mtKR Exposed to Light and IR

**Figure 4:**
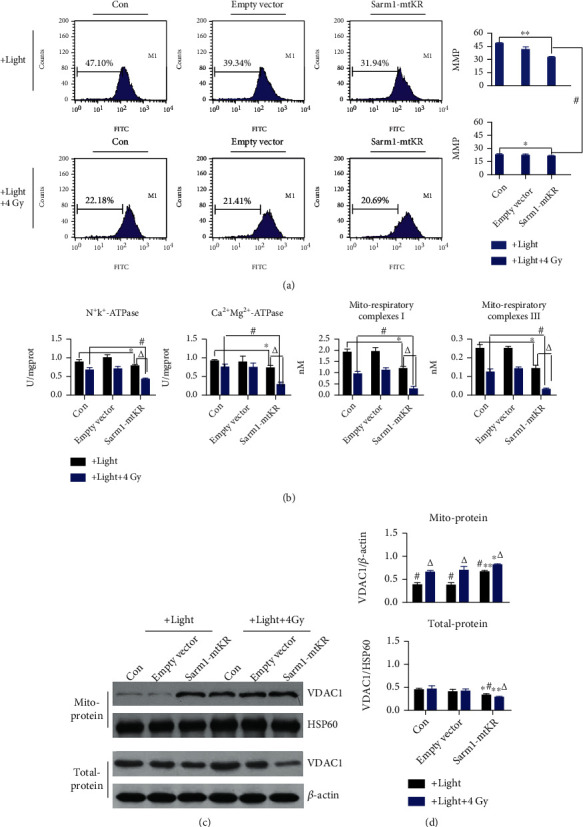
Mitochondrial dysfunction caused by mROS from Sarm1-KR and IR. (a) Representative FCM pictures of MMP in HeLa cells stained by Rh123 and analyzed by FCM. (b) The changes of Na^+^K^+^-ATPases, Ca^2+^Mg^2+^-ATPase, and mito-respiratory complex I and III activities by biochemical assay after light exposure and IR. (c) WB analysis was performed to determine protein levels of VDAC1 in total- and mito-protein. *β*-Actin and HSP60 proteins were used for loading control. (d) From top to bottom, the changes of VDAC1/*β*-actin and VDAC1/HSP60. Bars represent mean ± SD of triplicate measurements. ^∗^*P* < 0.05 and ^∗∗^*P* < 0.01, versus control; ^#^*P* < 0.05, versus 4 Gy IR; and ^△^*P* < 0.05, versus light exposure.

Furthermore, we explored the damage degree caused by mROS from Sarm1-KR exposed to light and IR, and MMP, Na^+^K^+^- and Ca^2+^Mg^2+^-ATPase, mito-respiratory complex I and III activities, and VDAC1 protein expression were measured. As shown in Figure 4(a), after Sarm1-mtKR plasmids were transfected into HeLa cells and then exposed to light, MMP significantly decreased (*P* < 0.01); at the same time, Na^+^K^+^- and Ca^2+^Mg^2+^-ATPase and mito-respiratory complex I and III activities also significantly decreased (*P* < 0.05, Figure 4(b)). Next, additional 4 Gy IR also had similar effects on HeLa cells, even enhanced the effects described as above. In addition, we found that VDAC1 expression in total protein decreased, while increased in mitochondrial protein in HeLa cells treated with Sarm1-mtKR and 4 Gy IR (Figures 4(c) and 4(d)). Taken together, these results indicated that mtKR and IR-induced ROS caused mitochondrial dysfunction, and mitochondrial permeability transition pore was kept in opening status.

### 3.5. Autophagy Caused by Sarm1-mtKR and IR

**Figure 5:**
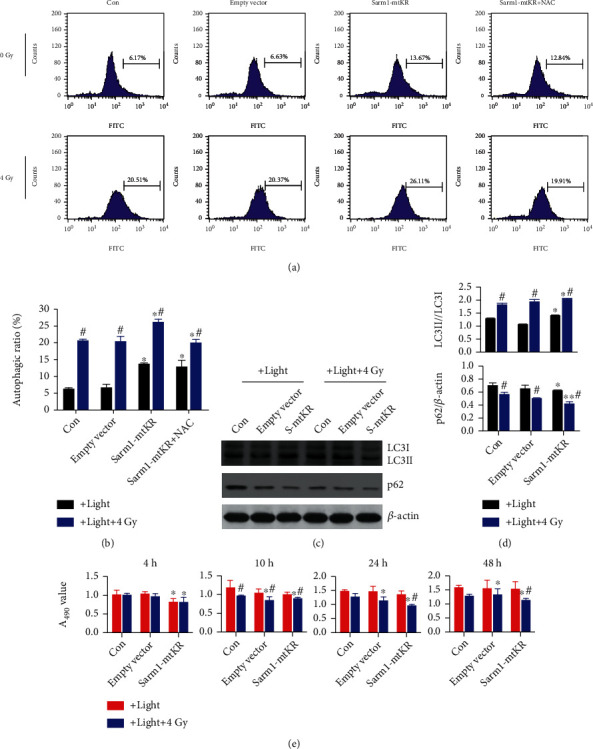
Sarm1-mtKR and IR promote autophagy and growth inhibition. (a) Representative pictures of autophagy detected by FCM in HeLa cells transfected with Sarm1-mtKR plasmids and followed NAC and IR treatment. (b) Statistical analysis of the autophagic rates was performed based on FCM data. (c) LC3 and p62 expression was detected by WB, and *β*-actin was used for loading control. (d) From top to bottom, the changes of LC3II/LC3I and p62/*β*-actin. (e) CCK8 assay demonstrates the inhibitory effects in HeLa cells transfected with Sarm1-mtKR plasmids and IR treatment. Bars represent mean ± SD of triplicate measurements. ^∗^*P* < 0.05 and ^∗∗^*P* < 0.01, versus control; ^#^*P* < 0.05, versus light exposure.

Autophagy has been shown to exhibit paradoxical function during cancer radiotherapy, to confer [29], or to overcome radioresistance [30, 31]. As shown in Figures 5(a) and 5(b), after Sarm1-mtKR plasmids were transfected into HeLa cells, light exposure of cells significantly increased autophagic rates as indicated by MDC staining (*P* < 0.05), and IR also resulted in a significant elevation of autophagic rates, but NAC could inhibit the effects. In addition, during the process of autophagy, the LC3I was converted to a lapidated form LC3II. Additionally, p62, an autophagy adaptor protein, also decreased, indicating the initiation of autophagy (Figures 5(c) and 5(d)). Next, the results of CCK8 showed that the cell growth was inhibited in Sarm1-mtKR-transfected HeLa cells exposed to light, and the inhibition was more obvious with combination of Sarm1-mtKR and IR (Figure 5(e)). These results indicated that elevation of autophagy might be the reason of growth inhibition induced by mROS from Sarm1-mtKR and IR.

### 3.6. Mitophagy Induced by mROS and IR Dependent on the Pink1/PARK2 Pathway

**Figure 6:**
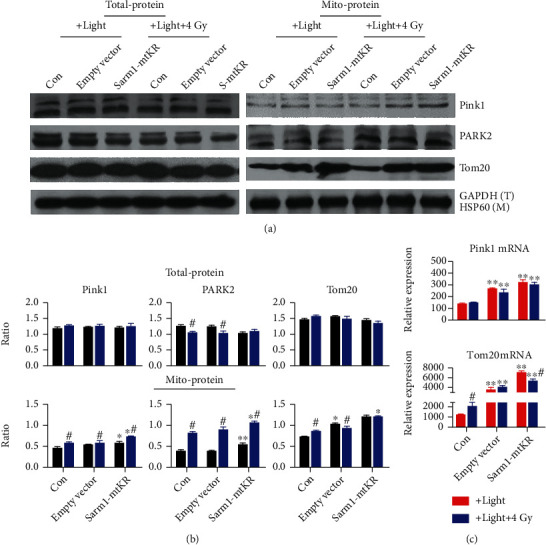
Sarm1-mtKR-induced mtROS participated in the autophagic cell death depending on the Pink1/PARK2 pathway. (a) WB was performed to determine protein levels of Pink1, PARK2, and Tom20 in total- and mito-proteins. GAPDH and HSP60 proteins were used for loading control. (b) The gray ratios of Pink1, PARK2, and Tom20 in total- and mito-proteins. (c) Pink1 and Tom20 mRNAs detected by qRT-PCR. Bars represent mean ± SD of triplicate measurements. ^∗^*P* < 0.05 and ^∗∗^*P* < 0.01, versus control; ^#^*P* < 0.05, versus light exposure.

Appropriate elimination of damaged and dysfunctional mitochondria plays a crucial role in preventing cellular further impairment from mtROS. Damaged mitochondria are mainly degraded via the mitochondrial selective autophagy machinery known as mitophagy [32]. Stress-caused membrane depolarization contributes to the recruitment of PARK2 to mitochondria, then to mediate ubiquitination of mitochondrial substrates. As shown in Figures 6(a) and 6(b), in total-protein, Pink1, PARK2, and Tom20 expressions showed no obvious changes, while all mito-protein expressions increased. Next, we found that mRNA expressions of Pink1 and Tom20 had similar regularity with mito-protein (Figure 6(c)). Therefore, we speculated that the Sarm1-mtKR expression in mitochondrial matrix could induce mtROS under visible light, then activate mitophagy depending on the Pink1/PARK2 pathway; particularly, combination of Sarm1-mtKR and IR could induce more autophagic death. Nevertheless, the hypothesis needs to be further verified by overexpression or inhibition of the Pink1/PARK2 pathway.

## 4. Discussion

Radiotherapy is the major means of cancer treatment but alone has limited curative and serious side effects, and overcoming radioresistance and enhancing radiosensitivity have become research hotspots. Traditionally, apoptosis is considered as the main death mode induced by IR during radiotherapy. However, more recent studies have suggested that autophagy is also important for IR-caused death, which may aid to improve radiosensitivity [7].

Autophagy is a highly conserved and regulated process of lysosomal degradation of organelles and long-lived protein macromolecules [33]. The deletion of autophagy-related proteins and genes promotes tumor initiation; moreover, tumor cells utilize it as a survival mechanism against the damage resulted from drug and IR. However, once autophagic death accumulates to a certain extent, autophagy plays an important anticarcinogenic role in the early stages of tumor initiation and in clearing damaged mitochondrial and aberrant protein [34, 35]. Recently, some studies reported that autophagy strengthens the anticancer effects of radiotherapy [4, 5]. In this study, we found that autophagic rate and LC3II/LC3I were significantly increased by IR in both HeLa and MCF-7 cells. In addition, we found that mtROS content and mitochondrial impairment were significantly elevated by IR; however, antioxidant NAC decreased the effects in MCF-7 cells, which is similar to another study [28].

As we know, mitochondria are sources of ROS, and accumulation of ROS has effects on membrane structure, to further cause mitochondrial dysfunction and then cell death. These results indicated that IR-induced autophagy is closely related to mitochondria and mtROS. ROS are a group of highly reactive chemicals under tight control of intracellular antioxidant [36]. In general, while antioxidant cancer therapy is justified by ROS in tumor initiation, promotion, and progression, prooxidant cancer therapy is also justified by the role of ROS in inducing apoptosis and reversing radioresistance [36]. An increasing body of documents has demonstrated that not only various therapeutic approaches depend on ROS but also further elevation of cellular ROS indeed can effectively kill more cancer cells [37, 38].

Exploiting the cancer cell killing potential of ROS could be performed by generation of ROS directly in tumor cells. Because of the direct harm of hydrogen peroxide to human health, great efforts have been made to find agents or treatments that stimulate endogenous ROS generation in tumor cells. Common drugs that increase cellular ROS include anthracyclines, platinum coordination complexes, topoisomerase inhibitors, and alkylating agents [39]. Therefore, how to induce enough ROS to target mitochondria is a crucial research topic in cancer treatment. KR is a genetically encoded red fluorescent protein. Under appropriate light excitation, KR can efficiently produce ROS that kill tumor cells and can be used for the inactivation of light-induced protein, killing specific cell populations *in vivo*, and studying intracellular local oxidative stress [24, 25, 40]. It will be a potential target of antitumor based on oxidative damage. In order to strengthen the mitochondrial targeting, the N-terminal 27 amino acids of Sarm1 act as a mitochondrial targeting signal sequence, and our results showed that recombinant fusion expression vector localized mitochondria was successfully constructed. Furthermore, the data showed that mtKR might promote mitochondrial ROS generation, therefore offering a significant opportunity to explore the cellular response upon ROS stress and to play a role of radiosensitization on HeLa cells.

Additionally, some studies demonstrated that IR exposure resulted in persistent accumulation of ROS [41, 42]. ROS might induce mitochondrial selective autophagy-lysosomal degradation termed mitophagy. The superoxide (O_2_^·−^) produced in the mitochondria has been previously shown to upregulate the formation of autophagosomes and subsequent catabolism upon autophagosomal fusion with lysosomes [43–45]. Thus, mitophagy plays an important role in mitochondrial quality control following stresses such as starvation, photo damage, and ROS production [46]. Mitochondrial dysfunction plays a key role in oxidative stress, and mtROS generation impairs the mitochondrial electron transport chain [47]. It is postulated that mitochondrial dysfunction in cancer cells would affect ATPase and mito-respiratory complex activities, and subsequent loss of MMP. Our results showed that after Sarm1-mtKR transfection and IR, indicators of mitochondrial dysfunction described as above all decreased, and the combination had a much stronger effect, while antioxidant NAC reduced the effects of Sarm1-mtKR on HeLa cells. At the same time, we also found that autophagic death and LC3II/LC3I increased, and p62 protein expression reduced; moreover, HeLa cell growth was inhibited. These results indicated the relationship among proliferation inhibition, mtROS, mitochondrial dysfunction, and mitophagy.

Currently, the most studied pathway of mitophagy initiation involves serine-threonine kinase Pink1 and E3 ubiquitin ligase PARK2. When mitochondria become damaged and MMP declines, the accumulation of Pink1 on the mitochondrial surface induced translocation of PARK2 from the cytosol to the damaged mitochondria, and then, the recruited PARK2 promoted the degradation of mitochondria through mitophagy [16]. Activated PARK2 causes ubiquitination of mitochondrial proteins including VDAC1, Tom20, and LC3 [48, 49]. More recently, many studies have dissected key mechanistic steps of this Pink1/PARK2-dependent mitophagy. The p62 protein is not only responsible for clumping of mitochondria through binding to PARK2-ubiquitylated mitochondrial substrates but also essential for PARK2 to promote mitophagy [48, 50]. In this study, we found that mROS caused by KR and IR enhanced Pink1/PARK2 protein expression in mitochondrial proteins. Indeed, some studies reported that Tom20, a mitophagy marker, is the primary binding partner of Pink1 and suggested to play a key role for accumulation of Pink1 and involve in the initial recognition of preproteins [51]. Here, we observed that Tom20 mRNA and protein expressions have similar regularity with Pink1. Taken together, our results demonstrated that the Pink1/PARK2 pathway might involve autophagy/mitophagy induced by the accumulation of mROS induced by mtKR and IR. Furthermore, we will perform the overexpression and inhibition of the Pink1/PARK2 pathway to verify the hypothesis. According to the results described as above, we summarized that IR might induce autophagic death of MCF-7 and HeLa cell, which might be related to the accumulation of mROS and mitochondrial impairment. Based on radiosensitization of cancer therapy depending on autophagy, we proposed that Sarm1-mtKR mediates ROS production on mitochondria, furthermore inducing mitochondrial dysfunction, autophagic death, and proliferation inhibition. Additionally, the mitophagy could be beneficial for radiosensitization of cancer depending on the Pink1/PARK2 pathway, and mitochondrial dysfunction and mitophagy have provided a new strategy for ROS sensitization. Next, the relative hypotheses will be further verified *in vivo* experiments, providing more sufficient data for clinical cancer therapy.

## Data Availability

In these studies, the data used to support the findings of this study are included within the article, all data were obtained by gene recombination technique, flow cytometry (FCM), biochemical assay, observation by fluorescence microscope, Western blot and quantitative real-time PCR, some pictures were plotted using different tools, such as Adobe Photoshop CS2 software, SPSS 24.0 version, GraphPad prism 6.0 software, and PPT of Microsoft office, Science-Slides, etc.
